# 308. Do lung ultrasound abnormalities change during hospitalization for COVID-19?

**DOI:** 10.1093/ofid/ofac492.386

**Published:** 2022-12-15

**Authors:** Jimin Hwang, Paul W Blair, Trishul Siddarthan, Gigi Liu, Erjia Cui, Jiawei Bai, Joshua East, Phabiola Herrara, Tiffany Fong, Varun Mahadevan, Shakir Hossen, Stefanie Seo, Olamide Sonuga, Joshua Lawrence, Lalaine Anova, Katherine Fenstermacher, Sophia Shea, Richard E Rothman, Bhakti Hansoti, Laruen Sauer, Ciprian Crainiceanu, Danielle Clark, Danielle Clark, Danielle Clark

**Affiliations:** Department of Epidemiology, Johns Hopkins Bloomberg School of Public Health, Baltimore, MD, Ellicott City, Maryland; The Henry M. Jackson Foundation for the Advancement of Military Medicine, Inc., Bethesda, MD; Division of Infectious Diseases, Johns Hopkins University School of Medicine, Baltimore, MD, Bethesda, Maryland; Division of Pulmonary and Critical Care Medicine, University of Miami, Miami, FL; Division of Pulmonary and Critical Care Medicine, Johns Hopkins University School of Medicine, Baltimore, MD, Miami, Florida; Division of General Internal Medicine, Johns Hopkins University School of Medicine, Baltimore, MD, Baltimore, Maryland; Department of Biostatistics, Johns Hopkins Bloomberg School of Public Health, Baltimore, MD, Baltimore, Maryland; Department of Biostatistics, Johns Hopkins Bloomberg School of Public Health, Baltimore, MD, Baltimore, Maryland; Division of Pulmonary and Critical Care Medicine, Johns Hopkins University School of Medicine, Baltimore, MD, Baltimore, Maryland; Division of Pulmonary and Critical Care Medicine, University of Miami, Miami, FL, Miami, Florida; Division of Pulmonary and Critical Care Medicine, University of Miami, Miami, FL, Miami, Florida; Division of Pulmonary and Critical Care Medicine, Johns Hopkins University School of Medicine, Baltimore, MD, Baltimore, Maryland; Division of Pulmonary and Critical Care Medicine, Johns Hopkins University School of Medicine, Baltimore, MD, Baltimore, Maryland; Department of Emergency Medicine, Johns Hopkins University, Baltimore MD, Baltimore, Maryland; Department of Emergency Medicine, Johns Hopkins University, Baltimore MD., Baltimore, Maryland; Department of Emergency Medicine, Johns Hopkins University, Baltimore MD., Baltimore, Maryland; The Henry M. Jackson Foundation for the Advancement of Military Medicine, Inc., Bethesda, MD, Bethesda, Maryland; Department of Emergency Medicine, Johns Hopkins University, Baltimore MD, Baltimore, Maryland; Department of Emergency Medicine, Johns Hopkins University, Baltimore MD, Baltimore, Maryland; John Hopkins University, Baltimore, Maryland; Department of Emergency Medicine, Johns Hopkins University, Baltimore MD, Baltimore, Maryland; Department of Emergency Medicine, Johns Hopkins University, Baltimore MD, Baltimore, Maryland; Department of Biostatistics, Johns Hopkins Bloomberg School of Public Health, Baltimore, MD, Baltimore, Maryland; The Henry M. Jackson Foundation for the Advancement of Military Medicine, Inc., Bethesda, MD, Bethesda, Maryland; The Henry M. Jackson Foundation for the Advancement of Military Medicine, Inc., Bethesda, MD, Bethesda, Maryland; The Henry M. Jackson Foundation for the Advancement of Military Medicine, Inc., Bethesda, MD, Bethesda, Maryland

## Abstract

**Background:**

While point-of-care ultrasound (POCUS) has been used to track disease resolution, temporal trends in lung ultrasound (LUS) findings among hospitalized patients with COVID-19 is not well-characterized.

**Methods:**

We studied 413 LUS scans in 244 participants ≥ 18 years of age hospitalized for COVID-19 pneumonia within 28 days of symptom onset from April, 2020 until September, 2021 at the Johns Hopkins Hospital, Baltimore Maryland. All patients were scanned using a 12-lung zone protocol and repeat scans were obtained in 3 days (N=114), 7 days (N=53), and weekly (N=9) from the initial scan. Participants were followed to determine clinical outcomes until hospital discharge and vital status at 28-days. Ultrasounds were independently reviewed for lung artifacts, and the composite mean LUS score (ranging from 0 to 3) across lung zones was determined. Trends of mean LUS scores and % lung fields with A-lines (indicating proportion of normal lung fields) were plotted by peak severity (mild, moderate, and severe defined by the World Health Organization Ordinal Scale) over time from symptom onset. Differences in mean LUS score or % A-lines changes over time between peak severity levels were evaluated using a Kruskal-Wallis test and linear mixed-effected models with an exchangeable correlation structure.

**Results:**

Among 244 patients in our cohort (mean age of 58.2 (SD 15.0) years, and 55.7% female) (Table 1), there was no change in average mean LUS scores between the first two visits by severity groups (Figure 1; Kruskal-Wallis p=0.63). Mean LUS scores were elevated by 0.22 (p< 0.001) in a dose-response manner regardless of duration of illness, but there was no change over time associated with peak severity (p=0.73). Similarly, percentage of A-lines were in 13.9% less lung fields for each increase in peak severity (p< 0.001; Figure 2) regardless of duration of illness. However, a change in mean LUS score did not differ significantly among peak severity levels (p=0.36).

**Figure:**
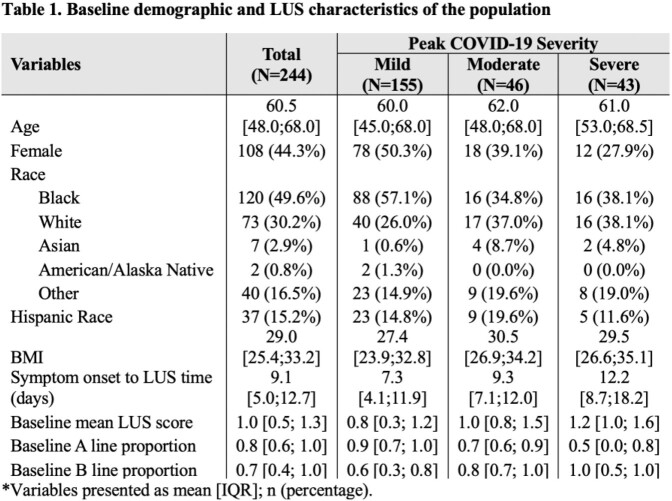

**Figure:**
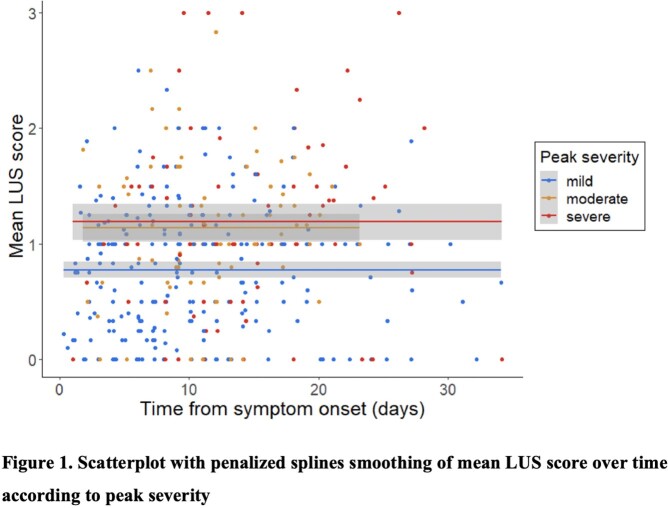

**Figure:**
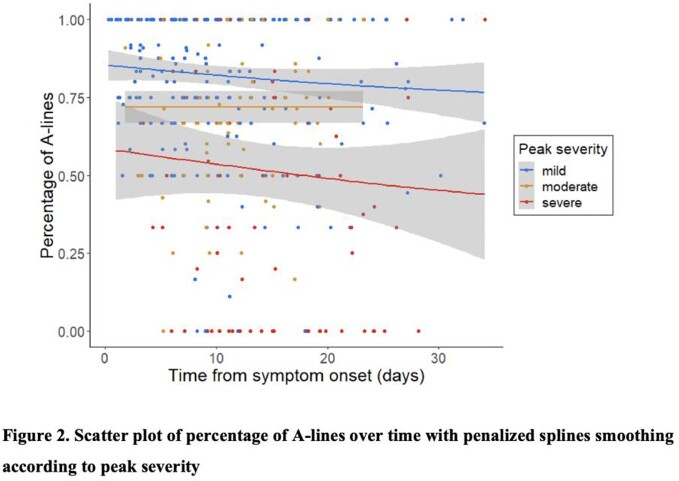

**Conclusion:**

Mean LUS scores correlated with clinical severity among hospitalized adults when assessed cross-sectionally, however mean LUS score did not change or differ between peak severity levels over the time course of hospitalization. These results do not support serial LUS scans to monitor disease progression.

**Disclosures:**

**All Authors**: No reported disclosures.

